# Patch-Clamp Recordings of Action Potentials From Human Atrial Myocytes: Optimization Through Dynamic Clamp

**DOI:** 10.3389/fphar.2021.649414

**Published:** 2021-04-12

**Authors:** Arie O. Verkerk, Gerard A. Marchal, Jan G. Zegers, Makiri Kawasaki, Antoine H. G. Driessen, Carol Ann Remme, Joris R. de Groot, Ronald Wilders

**Affiliations:** ^1^ Department of Medical Biology, Amsterdam UMC, University of Amsterdam, Amsterdam, Netherlands; ^2^ Department of Experimental Cardiology, Amsterdam UMC, University of Amsterdam, Amsterdam, Netherlands; ^3^ Department of Cardiothoracic Surgery, Amsterdam UMC, University of Amsterdam, Amsterdam, Netherlands; ^4^ Department of Cardiology, Amsterdam UMC, University of Amsterdam, Amsterdam, Netherlands

**Keywords:** drug testing, patch clamp, human, cardiac myocytes, left atrial appendage, action potential, dynamic clamp, inward rectifier potassium current

## Abstract

**Introduction:** Atrial fibrillation (AF) is the most common cardiac arrhythmia. Consequently, novel therapies are being developed. Ultimately, the impact of compounds on the action potential (AP) needs to be tested in freshly isolated human atrial myocytes. However, the frequent depolarized state of these cells upon isolation seriously hampers reliable AP recordings.

**Purpose:** We assessed whether AP recordings from single human atrial myocytes could be improved by providing these cells with a proper inward rectifier K^+^ current (I_K1_), and consequently with a regular, non-depolarized resting membrane potential (RMP), through “dynamic clamp”.

**Methods:** Single myocytes were enzymatically isolated from left atrial appendage tissue obtained from patients with paroxysmal AF undergoing minimally invasive surgical ablation. APs were elicited at 1 Hz and measured using perforated patch-clamp methodology, injecting a synthetic I_K1_ to generate a regular RMP. The injected I_K1_ had strong or moderate rectification. For comparison, a regular RMP was forced through injection of a constant outward current. A wide variety of ion channel blockers was tested to assess their modulatory effects on AP characteristics.

**Results:** Without any current injection, RMPs ranged from −9.6 to −86.2 mV in 58 cells. In depolarized cells (RMP positive to −60 mV), RMP could be set at −80 mV using I_K1_ or constant current injection and APs could be evoked upon stimulation. AP duration differed significantly between current injection methods (*p* < 0.05) and was shortest with constant current injection and longest with injection of I_K1_ with strong rectification. With moderate rectification, AP duration at 90% repolarization (APD_90_) was similar to myocytes with regular non-depolarized RMP, suggesting that a synthetic I_K1_ with moderate rectification is the most appropriate for human atrial myocytes. Importantly, APs evoked using each injection method were still sensitive to all drugs tested (lidocaine, nifedipine, E-4031, low dose 4-aminopyridine, barium, and apamin), suggesting that the major ionic currents of the atrial cells remained functional. However, certain drug effects were quantitatively dependent on the current injection approach used.

**Conclusion:** Injection of a synthetic I_K1_ with moderate rectification facilitates detailed AP measurements in human atrial myocytes. Therefore, dynamic clamp represents a promising tool for testing novel antiarrhythmic drugs.

## Introduction

Atrial fibrillation (AF) is the most common type of sustained cardiac arrhythmia in the elderly, with a substantially increasing prevalence anticipated over the next decades (Wakili et al., 2011; Milnes et al., 2012; Colilla et al., 2013; Krijthe et al., 2013). Data from the Framingham Heart Study revealed that AF is independently associated with a 50–90% increase in the risk of death (Benjamin et al., 1998). Clinical AF therapy focuses on prevention of severe complications secondary to AF, in addition to restoration of normal atrial rhythm (Milnes et al., 2012; Kirchhof et al., 2016; January et al., 2019). However, currently available drugs are associated with significant side effects as most of these compounds also affect ventricular function (Milnes et al., 2012). Consequently, current research focuses on atrial-specific pharmacological targeting of ion channels, such as K_V_1.5, Kir3.1/3.4, small conductance Ca^2+^-activated K^+^ (SK) channels, and Na_V_1.5, in order to generate new therapeutic strategies for AF (Antzelevitch and Burashnikov, 2010; Dobrev and Nattel, 2010; Wakili et al., 2011; Dobrev et al., 2012; Milnes et al., 2012; Peyronnet and Ravens, 2019).

Various *in vitro* (Papke and Smith-Maxwell, 2009; Ebert et al., 2012; Devalla et al., 2015; Hu et al., 2018) and *in vivo* animal models (Olgin and Verheule, 2002; Finet et al., 2009; Nishida et al., 2010), including AF models, are available for atrial drug testing, all with their individual strengths and limitations. While these models provide an excellent starting point for atrial and AF-related drug studies, assessments of action potentials (APs) of native human atrial cardiomyocytes remain essential due to distinct inter-species differences in ion channel expression profiles (Varró et al., 2021), and because drugs frequently act through AP shortening or prolonging effects, which may be frequency dependent (Peyronnet and Ravens, 2019). Samples of human left or right atrial appendage can be readily obtained during cardiac surgery (Hatem et al., 2010; Voigt et al., 2015), and studies employing such samples have provided detailed knowledge of human atrial electrophysiology in healthy and diseased states, as reviewed elsewhere (Nattel et al., 2007; Schotten et al., 2011; Dobrev et al., 2012; Heijman et al., 2014). So far, a variety of methods have been used for the electrophysiological analysis of human atrial tissue and cells, including sharp electrode measurements (Trautwein et al., 1962; Ten Eick and Singer, 1979), patch clamp methodology (Van Wagoner et al., 1997; Hoppe and Beuckelmann, 1998), multi-electrode arrays (MEAs) (Kanagaratnam et al., 2006), and voltage-sensitive fluorescence (Krul et al., 2015). In particular, the patch clamp technique, which is considered the gold standard in electrophysiology due to the ability to measure both membrane currents and APs in single cells (Kornreich, 2007), has demonstrated the functional presence of various types of ion channels in human atrial cells. However, AP measurements from isolated human atrial myocytes using patch clamp methodology are limited by the frequent depolarized state of the resting membrane potential (RMP) of these myocytes (Hoppe and Beuckelmann, 1998; Colman et al., 2018). Upon isolation, generally only a fraction of cells, approximately 5–20%, have an RMP sufficiently negative to elicit APs (Amos et al., 1996; Schreieck et al., 2000), and even within this fraction highly depolarized myocytes are frequently excluded from analysis (Loose et al., 2014).

Various patch clamp studies on human atrial myocytes have addressed this major problem of depolarized state by injection of a hyperpolarizing current of constant amplitude, resulting in an RMP near −80 mV (Wang et al., 1993; Le Grand et al., 1994; Bénardeau et al., 1996; Workman et al., 2001; Pau et al., 2003; Workman et al., 2003; Lagrutta et al., 2006; Limberg et al., 2011; Workman et al., 2012; Skibsbye et al., 2014; Schmidt et al., 2015). However, this hyperpolarizing current of constant amplitude also affects the AP repolarization phase to a considerable extent, thereby potentially hampering the assessment of the impact of drugs on AP duration (APD).

Human induced pluripotent stem cell-derived cardiomyocytes (hiPSC-CMs) frequently display similar depolarized membrane potentials due to their relative immaturity (Veerman et al., 2015). In these cells, however, the dynamic clamp methodology (Wilders, 2006; Berecki et al., 2014; Ortega et al., 2018; Verkerk and Wilders, 2021) has been successfully applied to overcome the depolarized state by injection of an *in silico* inward rectifier K^+^ current (I_K1_) (Bett et al., 2013; Meijer van Putten et al., 2015; Verkerk et al., 2017), which is the main current responsible for setting the RMP close to the potassium equilibrium potential (E_K_) of approximately −85 mV. Consequently, hiPSC-CMs could be stimulated at fixed rates with fast sodium current (I_Na_) driven upstrokes. Furthermore, this dynamic clamp reduced the variability of various AP parameters (Verkerk et al., 2017), thus allowing easier detection of small changes in these parameters.

We here tested whether the injection of a synthetic I_K1_, which is computed in real time based on the acquired membrane potential and then injected into the cell through the patch pipette, can also improve AP measurements in human left atrial appendage (LAA) myocytes. We used two different I_K1_ current-voltage relationships, i.e., one with large and one with moderate rectification, and compared the results to those obtained with the injection of a repolarizing current of constant amplitude. In addition, we tested a broad range of ion channel blockers to see if drugs are still able to modify AP characteristics under these conditions and to test if drug effects are dependent on the current injection approach used.

## Materials and Methods

### Human Cell Preparation

#### Human Myocardial Tissue

Written informed consent was obtained from all patients, and the study was approved by the local ethics committee and conducted in accordance with the Declaration of Helsinki. LAAs were obtained from eight patients with paroxysmal AF undergoing minimally invasive surgical ablation (mini-maze procedure). Table 1 shows the patient characteristics. Immediately after surgical removal of the specimens, the LAA tissue was placed in chilled (0°C) Ca^2+^-free MOPS solution, and carried to the laboratory within 30 min. The Ca^2+^-free MOPS solution was composed of (in mM): 100 NaCl, 10 KCl, 5 glucose, 5 MgSO_4_, 50 taurine, 11 creatine, 5 MOPS; pH 7.0 (NaOH).

**TABLE 1:** Patient characteristics.

Number of patients, *n*	8
Gender, M/F	6/2
Age (years)	53.3 ± 3.4
Heart rate (beats min^−1^)^a^	58.4 ± 4.0
PR interval (ms)^a^	179.5 ± 10.2
QRS interval (ms)^a^	102.0 ± 9.4
QTc interval (ms)^a^	431.0 ± 9.2
Hypertension, *n*	5
Pulmonary hypertension, *n*	1
Diabetes, *n*	N/A
ACE inhibitors, *n*	6
Ca^2+^ antagonists, *n*	1
Class IC blockers, *n*	4
Class II blockers, *n*	2
Class III blockers, *n*	5
Lipid-lowering drugs, *n*	4

^a^ In sinus rhythm. ACE, angiotensin-converting enzyme.

#### Isolated Human Atrial Myocytes

Single cells were obtained by an enzymatic isolation method modified from Dobrev et al. (2000). In short, fatty tissue and endocardial connective tissue were removed and the remaining tissue was cut into small cubic pieces (≈1 mm^3^), and washed three times for 10 min in Ca^2+^-free MOPS solution (37°C). Then, the LAA tissue pieces were incubated for 60 min in Ca^2+^-free MOPS solution (37°C) to which collagenase A (250 U/ml; Roche) and proteinase XXIV (3.5–7.0 U/ml; Sigma) were added. In these 60 min, the Ca^2+^ concentration was gradually increased to 0.15 mM by adding Ca^2+^ three times (i.e., at 15, 40, and 50 min). Subsequently, the tissue pieces were placed in 0.1 mM Ca^2+^ MOPS solution (37°C) with collagenase A (310 U/ml; Roche). During this last incubation period, the tissue pieces were gently stirred and at regular intervals of 15 min the solution was microscopically examined for the presence of dissociated rod-shaped myocytes. Once single, rod-shaped myocytes were observed (usually after 75 min), cells were removed, and this was repeated every 10 min for 80 min, resulting in several batches of isolated cells. The isolated cells were transferred into Ca^2+^-free MOPS solution (37°C) containing 1% BSA. Isolated cells were stored for at least 45 min at room temperature in a modified Tyrode’s solution containing (in mM): 145 NaCl, 5.4 KCl, 0.9 CaCl_2_, 0.5 MgCl_2_, 5.5 glucose and 5.0 HEPES; pH 7.4 (NaOH).

### Electrophysiological Experiments

#### Recording Procedures and Data Acquisition

The myocytes were placed in a cell chamber mounted on the stage of an inverted microscope (Nikon Diaphot), and allowed to settle for 5 min before they were superfused with Tyrode’s solution containing 1.8 mM CaCl_2_ and 1 mM MgCl_2_. Single myocytes having smooth surfaces with cross striations were selected for electrophysiological measurements. APs were recorded using an Axopatch 200B amplifier (Molecular Devices, Sunnyvale, CA, United States). Data acquisition, voltage control, and analysis were accomplished using custom software. All potentials were corrected for the estimated liquid junction potential (Barry and Lynch, 1991). Pipettes (resistance 2–3 MΩ) were pulled from borosilicate glass capillaries (Harvard Apparatus, United Kingdom) using a custom-made vertical microelectrode puller. Cell membrane capacitance (C_m_) was estimated by dividing the time constant of the decay of the capacitive transient in response to 5 mV hyperpolarizing voltage clamp steps from −40 mV by the series resistance. Average C_m_ was 114 ± 9 pF (*n* = 58; mean ± SEM). Signals were low-pass filtered with a cut-off frequency of 5  kHz and digitized at 40  kHz.

#### Action Potentials

APs were measured at 36 ± 0.2°C using the amphotericin-perforated patch-clamp method. These were mostly obtained from the 5–8th isolation batch of myocytes, because the myocytes of the first batches had relatively “weak” membranes and frequently displayed loss of seal, unwanted ruptured patch, and/or large “leak currents”. Pipette solution contained (in mM): 125  K-gluc, 20 KCl, 5 NaCl, 0.44 amphotericin-B, 10 HEPES (pH 7.2; KOH). APs were continuously elicited at 1 Hz with square 3-ms, ≈1.2× threshold current pulses through the patch pipette. APs were measured at steady-state baseline conditions and at 5 min after drug application. We analyzed RMP, maximum AP upstroke velocity (dV/dt_max_), AP amplitude (APA), AP plateau amplitude (PlatA; measured 20 ms after initiation of the AP upstroke), and APD at 20, 50, and 90% repolarization (APD_20_, APD_50_, and APD_90_, respectively). Parameters from 10 consecutive APs were averaged.

#### Constant Current Injection and Dynamic Clamp

**FIGURE 1:**
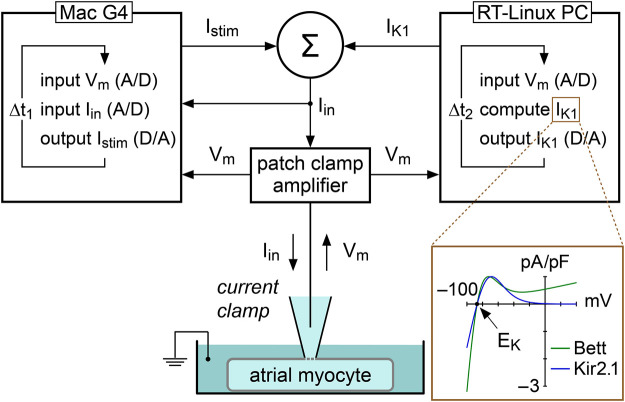
Experimental approach to supply a patched atrial myocyte with a synthetic inward rectifier K^+^ current (I_K1_). A Real-Time Linux (RT-Linux) based PC computes the synthetic I_K1_ according to the membrane potential (V_m_) that is read into the PC. After addition of any stimulus current (I_stim_), this I_K1_ is sent to the patch clamp amplifier, which operates in current clamp mode and injects the net current (I_in_) into the patched myocyte. This process is updated with a time step Δt_2_ of 40 µs. I_stim_ is sent out by the Apple Macintosh (Mac) G4 computer that is used to control the experiment and record both V_m_ and I_in_ at a rate of 40 kHz, corresponding with a time step Δt_1_ of 25 µs. Inset: current–voltage relationship of the synthetic I_K1_ with moderate rectification (“Bett” current; Bett et al., 2013) and the synthetic I_K1_ with strong rectification (“Kir2.1” current; Meijer van Putten et al., 2015). E_K_ denotes the potassium equilibrium potential.

In the present study, we used constant and dynamic clamp current injections to set the RMP of single atrial myocytes at −80 mV, i.e., close to the RMP in atrial tissue from patients with AF (Christ et al., 2008). Constant current was injected with our standard patch clamp software and the amount of injected current was adapted in every myocyte to set its RMP at −80 mV. A custom dynamic clamp setup was used to inject an *in silico* I_K1_, as illustrated in Figure 1, and previously described in detail (Meijer van Putten et al., 2015). In short, we extended our standard patch clamp setup with a separate Real-Time Linux (RT-Linux) based PC that continuously reads in the membrane potential (V_m_) of the atrial myocyte and computes the V_m_-dependent synthetic I_K1_. Within the time step ∆t_2_ of 40 µs, a command potential is generated that, combined with a command potential for any stimulus current, is sent to the patch clamp amplifier to inject this current into the human atrial myocyte. For our synthetic I_K1_, we used two different I_K1_ current-voltage relationships.

One I_K1_ had a current-voltage relationship based on a fit to data from Kir2.1 channels expressed in HEK-293 cells by Dhamoon et al. (2004), defined “Kir2.1” in the remainder of the study. The Kir2.1 current has a strong rectification, as illustrated in Figure 1 (inset), resulting in absence of a substantial current positive to −20 mV (Dhamoon et al., 2004; Meijer van Putten et al., 2015), and is computed according to:

$$I_{K1}=A\times 0.12979\times \left\{\frac{V_{m}-E_{K}}{1.0+\;e^{\left[0.093633\;\times \left(V_{m}+72\right)\right]}}\right\},$$

in which V_m_ and E_K_ denote the membrane potential and potassium equilibrium potential (in mV), respectively, and E_K_ amounts to −86.9 mV. “A” denotes a scaling factor which is adapted in every experiment to set the RMP at −80 mV. If A is set to 1, the peak outward current density of I_K1_ amounts to 1 pA/pF.

The other I_K1_ had a current-voltage relationship showing moderate rectification, defined “Bett” in the rest of the study, referring to the I_K1_ used in the dynamic clamp study by Bett et al. (2013). Their I_K1_ has a substantial current at positive potentials, as illustrated in Figure 1 (inset), consistent with I_K1_ currents observed in human atrial myocytes (Koumi et al., 1995), and is computed according to:

$$I_{K1}=A\;\times \;\left[0.307\;\times \left(\frac{V_{m}-E_{K}}{1+e^{\left(0.0896\left(V_{m}-E_{K}\right)\right)}}\right)+0.00614\;\times \left(V_{m}-E_{K}\right)\right],$$

in which V_m_ and E_K_ denote the membrane potential and potassium equilibrium potential (in mV), respectively, and E_K_ amounts to −86.9 mV. “A” denotes a scaling factor which is adapted in every experiment to set the RMP at −80 mV. If A is set to 1, the peak outward current density of *I*_K1_ equals 1 pA/pF.

### Drugs

Various general ion channel blockers with well-known effects on human atrial tissue and/or cells were used to test their ability to modify AP characteristics when applying current injection and to validate if potential drug effects depend on the I_K1_ or constant current injection approach used. Each drug was tested in myocytes isolated from two patients. 4-Aminopyridine (4-AP; Merck) was freshly prepared as 10 mM stock solution in Tyrode’s solution and pH was readjusted to 7.4 (HCl). Nifedipine (Sigma) was prepared as 5 mM stock solution in ethanol, and stored in the dark. E-4031 (Tocris), apamin (Sigma), and BaCl_2_ (Sigma) were prepared as 5, 0.1, and 1000 mM stock solutions, respectively, in distilled water. All stock solutions and lidocaine (10 mg/ml; Braun) were diluted appropriately before use.

### Statistics

All data are presented as mean ± SEM. Potential differences in membrane capacitance between non-depolarized and depolarized myocytes were assessed with an unpaired Student’s *t*-test. Drug effects were tested with a paired Student’s *t*-test. Differences between three or more groups were assessed using one-way RM ANOVA followed by pairwise comparison using the Student-Newman-Keuls test. *p* < 0.05 was considered statistically significant.

## Results

### Basic Action Potential Characterization of Isolated Human Atrial Myocytes

**FIGURE 2:**
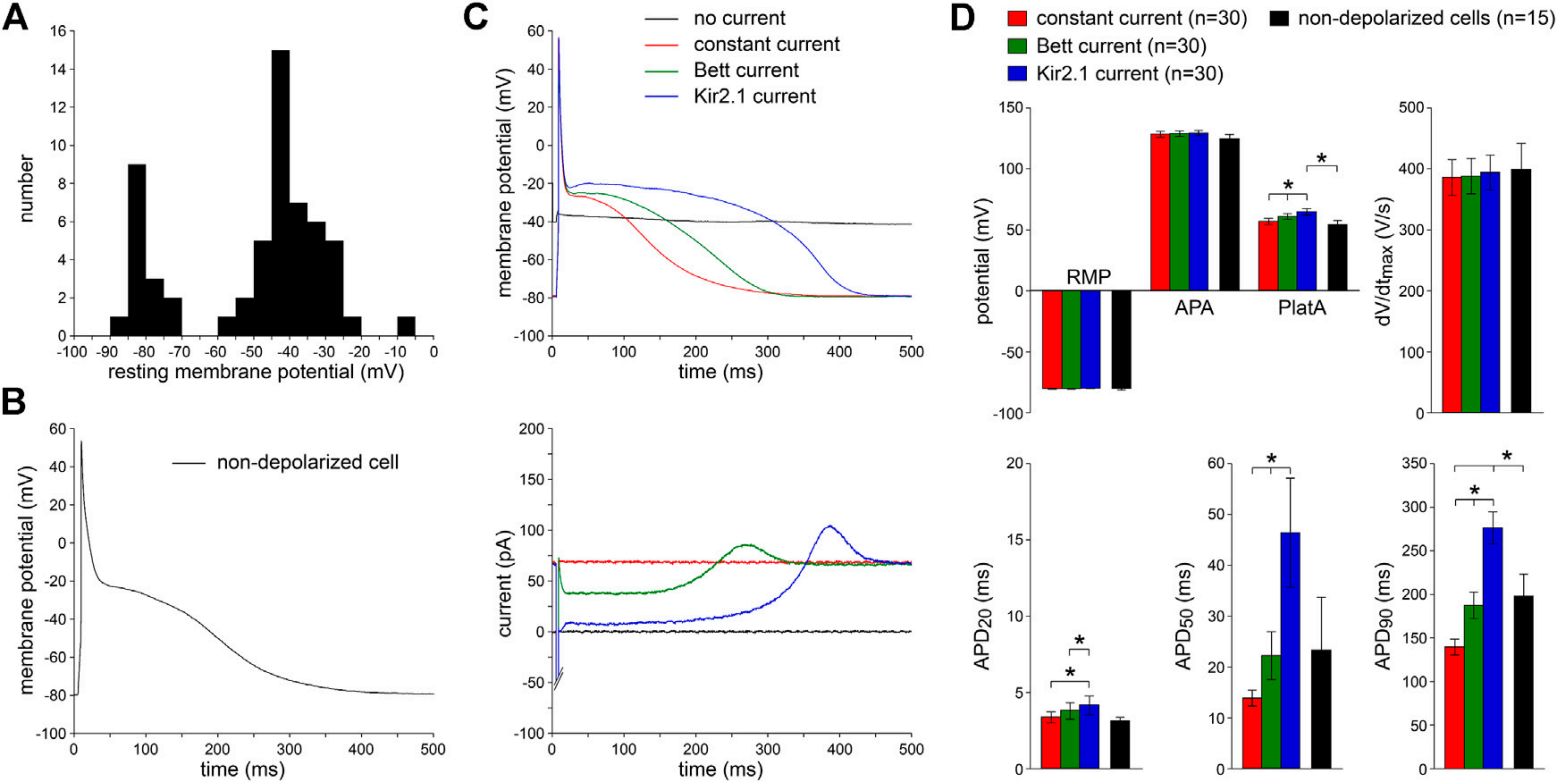
Effects of injection of constant repolarizing current or synthetic I_K1_ (with moderate or strong rectification, supplied through dynamic clamp) on action potentials of enzymatically isolated human atrial myocytes. **(A)** Resting membrane potential (RMP) of 58 human atrial myocytes in absence of repolarizing current injection. Note the group of 15 non-depolarized myocytes (leftmost group of bars) with an RMP of −70 mV or more negative. **(B)** Typical action potential (AP), elicited at 1 Hz, of a non-depolarized myocyte in absence of any repolarizing current injection. **(C)** **Top:** typical APs elicited at 1 Hz in presence of injected constant or I_K1_ current within one originally depolarized cell (with an original RMP near −40 mV). **Bottom:** associated injected currents. Synthetic I_K1_ supplied as detailed in Figure 1. **(D)** Average AP parameters of 30 originally depolarized myocytes during constant current and I_K1_ injections with dynamic clamp. Non-depolarized cells are the 15 cells of panel A with an RMP of −70 mV or more negative in absence of any repolarizing current injection. APA, AP amplitude; PlatA, amplitude of the AP plateau; dV/dt_max_, maximum AP upstroke velocity; APD_20_, APD_50_, and APD_90_, AP duration at 20, 50, and 90% repolarization, respectively. *p < 0.05.

We patched 58 single human atrial myocytes enzymatically isolated from LAAs that were obtained from eight patients with paroxysmal AF (Table 1). Figure 2A shows the distribution of the RMP of these cells. In only 15 (26%) of these cells, we were able to obtain an RMP sufficiently negative (−70 mV or more negative; Figure 2A, leftmost group of bars) to elicit APs. A typical AP of an intrinsically non-depolarized myocyte is shown in Figure 2B. C_m_ did not differ significantly between non-depolarized and depolarized myocytes (112 ± 13 vs. 115 ± 12 pF, respectively) and the AP parameters of the 15 intrinsically non-depolarized cells appear in Figure 2D as black bars. The remaining 43 (74%) of these cells were too depolarized to evoke APs with an I_Na_ driven upstroke (Gelband et al., 1972; Berecki et al., 2010). Typically, the RMP of these cells was positive to −60 mV (Figure 2A, rightmost group of bars).

### Effects of I_K1_ and Constant Current Injection on Action Potentials

Next, we studied the effects of the two types of dynamic clamp-injected synthetic I_K1_ currents (Figure 1, inset) as well as constant current injection on the AP morphology of 30 depolarized myocytes. To compare potential differences of these three approaches, all types of current injection were tested in every cell measured. Figure 2C, top panel, shows representative recordings of the membrane potential. The associated traces in Figure 2C, bottom panel, show the injected currents. In the absence of I_K1_ or constant current injection, the membrane potential was around −40 mV and no APs could be evoked (black lines). Upon switching on the dynamic clamp system with the synthetic “Kir2.1” I_K1_ [i.e., the I_K1_ with strong rectification (Figure 1, inset)], the RMP hyperpolarized consistent with the generated outward current (blue lines). We chose an I_K1_ amplitude that set the RMP at −80 mV, which is close to the RMP in human AF cells previously reported by Christ et al. (2008) and to the RMP that we observed in the 15 intrinsically non-depolarized cells (Figure 2D). At this potential, the synthetic I_K1_ was still substantial [0.71 ± 0.08 pA/pF (*n* = 30), consistent with the current-voltage relationship of Kir2.1]. When the synthetic I_K1_ was added, APs could be elicited, and average AP characteristics with the Kir2.1 injection are summarized Figure 2D (blue bars). The APs had fast upstroke velocities of ≈400 V/s, indicating that they were importantly driven by I_Na_ (Berecki et al., 2010). The phase 1 repolarization was fast, resulting in a relatively short APD_20_ and a plateau potential of around −20 mV, typical for human atrial myocytes with the co-called “Type A” (Bénardeau et al., 1996) and “Type 3” (Dawodu et al., 1996; Workman et al., 2001) AP shape. Thus, the dynamic clamp-injected Kir2.1 I_K1_ enabled the generation of APs in originally depolarized human atrial myocytes.

We followed the same approach for the injection of the “Bett” I_K1_, i.e., the I_K1_ with moderate rectification (Figure 1, inset). The results are shown in Figure 2C,D, as green lines and bars, respectively. Again, the RMP was successfully hyperpolarized to −80 mV [with an I_K1_ amplitude of 0.73 ± 0.08 pA/pF (*n* = 30) at this potential], and APs could be evoked. However, the APD was significantly shorter at all repolarization phases and the AP plateau amplitude was significantly lower compared to the APs with Kir2.1 I_K1_ injection (Figure 2C,D). As expected from the I_K1_ current-voltage relationships (Figure 1, inset), this was due to the lower amount of rectification resulting in a larger injected outward current during phase 1 and phase 2 of the AP (Figure 2C, bottom). In each of the cells, we also applied the constant current injection (Figure 2C,D, red lines and bars). Again, the RMP was successfully hyperpolarized to −80 mV [with an outward current amplitude of 0.71 ± 0.09 pA/pF (*n* = 30)]. However, this approach was accompanied by a severe AP shortening compared to APs measured with Kir2.1 I_K1_ injection (Figure 2C,D), due to the large injected outward current over the complete course of the AP (Figure 2C, bottom).

While all three types of injected current resulted in successful RMP hyperpolarization to −80 mV, and consequently allowed the elicitation of I_Na_ driven APs, the APD and AP plateau amplitude differed significantly between the methods used. On average, APD_20_, APD_50_, and APD_90_ were longest using the Kir2.1 current and shortest with the constant current injection (Figure 2D). Neither the APA nor the dV/dt_max_ differed between the three different current types, but AP plateau amplitude was lowest with constant current injection and highest with injection of Kir2.1 (Figure 2D). To test which approach most closely resembled the physiological conditions, we compared the AP parameters measured with each of the three types of current injection with those of the non-depolarized cells. As shown in Figure 2D, the average AP parameters of the 15 non-depolarized cells (black bars) matched closely with the AP parameters measured with the current injection methods, except that APD_90_ significantly differed from the Kir2.1 and constant current injection approaches. In addition, the AP plateau amplitude was significantly different between the non-depolarized cells and the cells injected with Kir2.1 I_K1_.

In summary, both I_K1_ formulations as well as the constant current injection appeared sufficient to obtain a stable RMP near −80 mV, enabling the generation of I_Na_ driven APs. Especially APs obtained with the Bett I_K1_ were highly similar to those of non-depolarized cells.

### Effects of Drugs

Next, we tested a wide range of ion channel blockers to find out whether these still exert their effects when applying current injection to obtain an RMP near −80 mV and whether the effects on AP morphology depend on the injection approach used. We anticipated that drug effects, especially those related to APD, may differ between approaches consequent to the observed APD differences among the latter (Figure 2D).

#### Lidocaine

**FIGURE 3:**
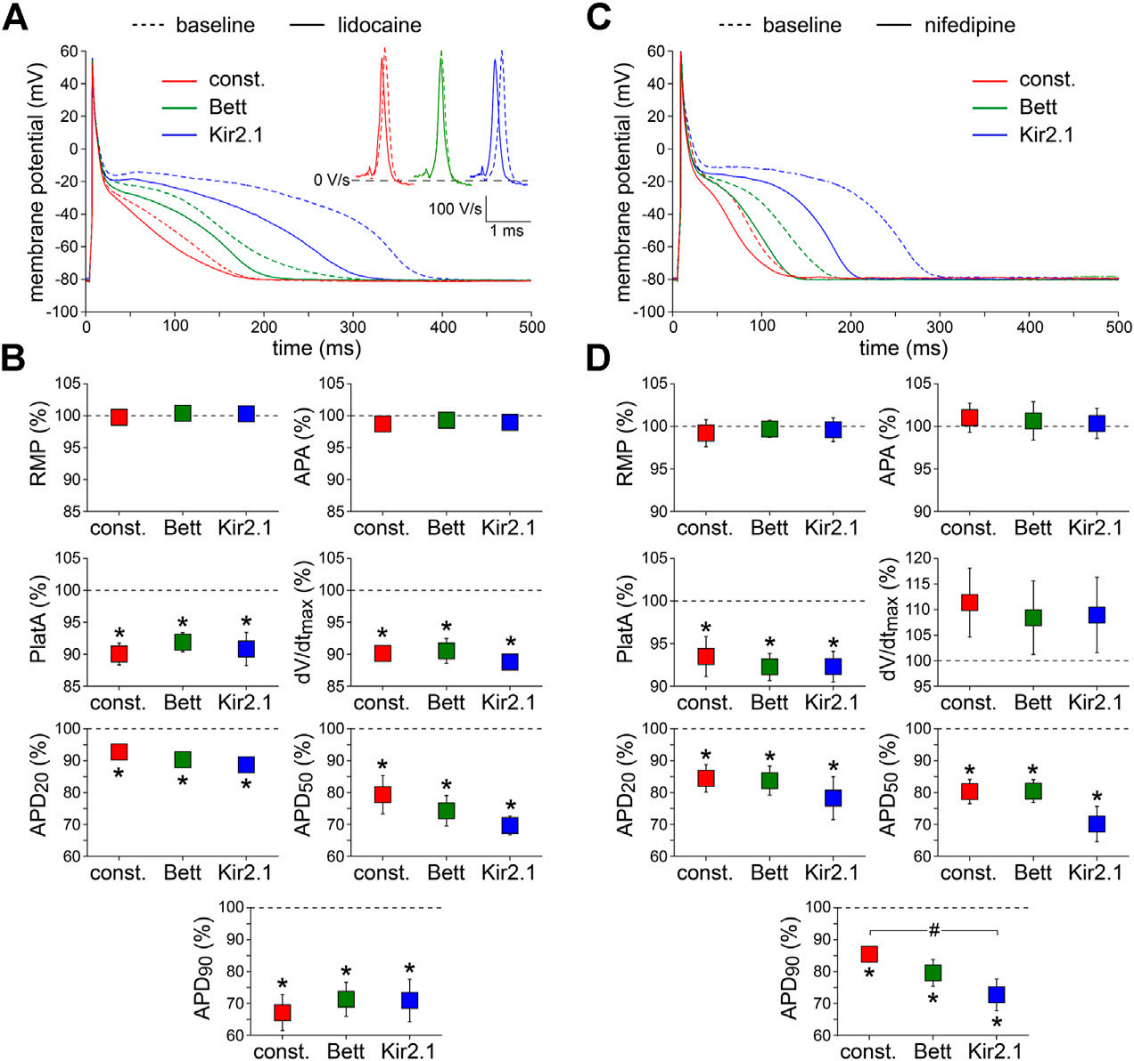
Effects of inward current blockade on APs measured with constant and I_K1_ current injection. **(A)** Typical APs of one atrial myocyte in absence (baseline) and presence of lidocaine to block the fast Na^+^ current. Inset: first derivatives of the AP upstrokes, reflecting the amplitude of the fast Na^+^ current. **(B)** Average AP parameters measured in five cells in presence of lidocaine. AP parameters are normalized to the AP parameters measured under baseline conditions in the same cell. **(C)** Typical APs of one atrial myocyte in absence (baseline) and presence of nifedipine to block the L-type Ca^2+^ current. **(D)** Average AP parameters measured in six cells in presence of nifedipine. AP parameters are normalized to the AP parameters measured under baseline conditions in the same cell. *p < 0.05, drug vs. baseline (paired *t*-test); ^#^ *p* < 0.05, drug effects observed with constant current, Bett current, and Kir2.1 current (ANOVA with follow-up test).

First, we tested the effects of 20 µM lidocaine, an antiarrhythmic agent that specifically blocks I_Na_ (Bean et al., 1983). Figure 3, shows typical examples (Figure 3A) and the average effects (Figure 3B) of the partial block of I_Na_ at this concentration of lidocaine, respectively. Lidocaine significantly reduced dV/dt_max_ by ≈ 10%, as also illustrated in Figure 3A, inset, and significantly shortened APD at all repolarization phases. AP plateau amplitude was significantly decreased, but RMP and APA were unaltered. The effects of lidocaine did not differ significantly between the I_K1_ and constant current injections.

#### Nifedipine

Second, 0.1 µM nifedipine was used to specifically block the L-type Ca^2+^ current (I_Ca,L_) by ≈ 50% (Eroglu et al., 2020). Figure 3C,D, show representative examples and summarizes the average effects on AP parameters, respectively. Nifedipine did not affect RMP, APA, or dV/dt_max_, but significantly reduced APD at all repolarization phases as well as AP plateau amplitude. The nifedipine-induced AP shortening was most prominent with injection of Kir2.1 I_K1_ (Figure 3C,D), i.e., the approach with the longest baseline APs. In case of APD_90_, the effects of nifedipine differed significantly between the Kir2.1 I_K1_ and constant current injection approaches (Figure 3D).

#### 4-Aminopyridine

**FIGURE 4:**
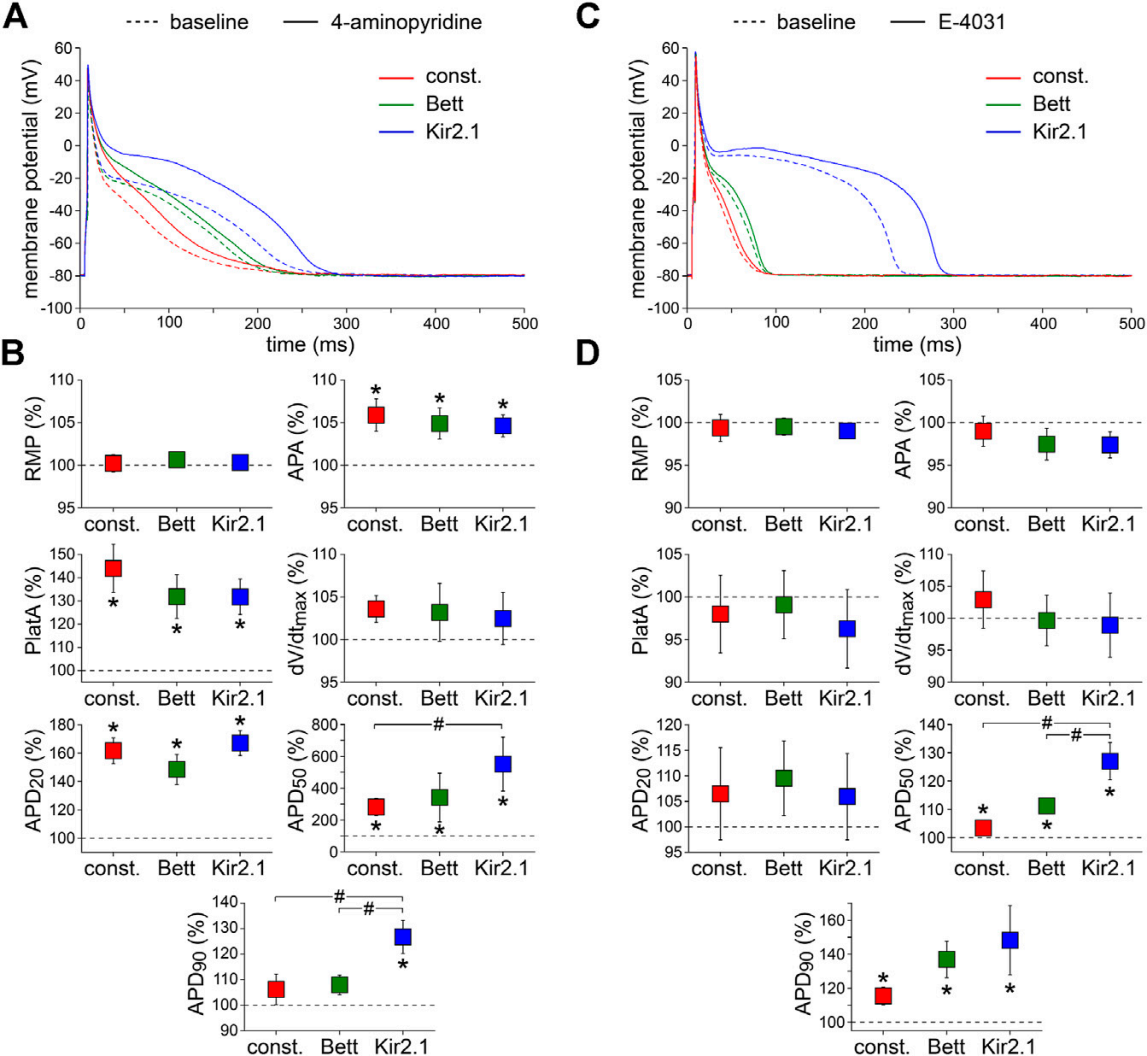
Effects of delayed rectifier K^+^ current blockade on APs measured with constant and I_K1_ current injection. **(A)** Typical APs of one atrial myocyte in absence (baseline) and presence of low dose 4-aminopyridine (4-AP) to block the ultra-rapid delayed rectifier K^+^ current. **(B)** Average AP parameters measured in five cells in presence of 4-AP. AP parameters are normalized to the AP parameters measured under baseline conditions in the same cell. **(C)** Typical APs of one atrial myocyte in absence (baseline) and presence of E-4031 to block the rapid delayed rectifier K^+^ current. **(D)** Average AP parameters measured in five cells in presence of E-4031. AP parameters are normalized to the AP parameters measured under baseline conditions in the same cell. *p < 0.05, drug vs. baseline (paired *t*-test); ^#^ *p* < 0.05, drug effects observed with constant current, Bett current, and Kir2.1 current (ANOVA with follow-up test).

Third, we assessed the effects of 50 µM 4-AP, which is known to reduce the ultra-rapid component of the delayed rectifier K^+^ current (I_Kur_) by ≈50% without affecting the transient outward K^+^ current (I_to1_) (Wang et al., 1993). Figure 4A,B, show typical examples of APs and the average effects of the low dose 4-AP, respectively. 4-AP affected all AP parameters except RMP and dV/dt_max_. The 4-AP-induced blockade of I_Kur_ did not only result in a significant increase in APA and AP plateau amplitude, but also in a significant AP prolongation at most repolarization phases. At 50% repolarization, the increase in APD was significantly larger with the Kir2.1 I_K1_ compared to the constant current injection method, and at 90% repolarization the increase in APD was significantly larger with the Kir2.1 I_K1_ compared to both the Bett I_K1_ and constant current injection methods.

#### E-4031

Fourth, we measured the effects of specific and complete blockade of the rapid component of the delayed rectifier K^+^ current (I_Kr_), which was induced by 5 µM E-4031 (Sanguinetti and Jurkiewicz, 1990). Figure 4C,D show typical examples and the average effects of E-4031, respectively. E-4031 resulted in a significant increase in both APD_50_ and APD_90_, without affecting other AP parameters. The effects of E-4031 on APD_50_ were most prominent with the Kir2.1 I_K1_ approach, resulting in a significantly larger increase as compared to the Bett I_K1_ and constant current injection methods.

#### Apamin

**FIGURE 5:**
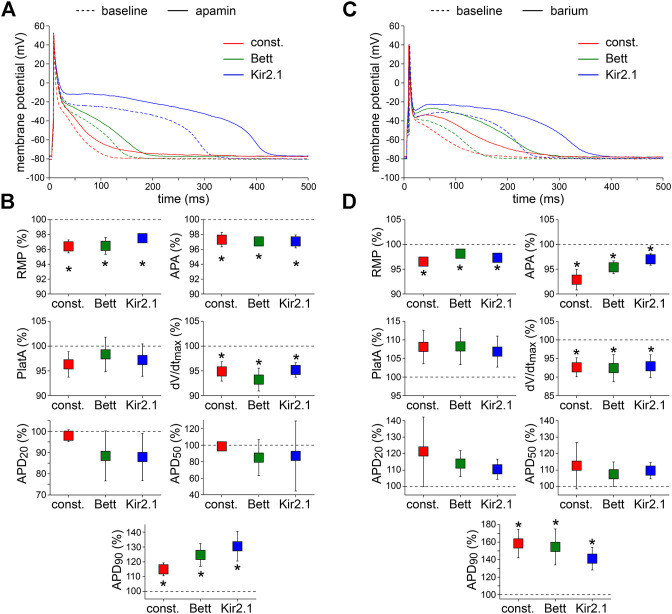
Effects of apamin and barium on APs measured with constant and I_K1_ current injection. **(A)** Typical APs of one atrial myocyte in absence (baseline) and presence of apamin to block the Ca^2+^-dependent K^+^ current. **(B)** Average AP parameters measured in five cells in presence of apamin. AP parameters are normalized to the AP parameters measured under baseline conditions in the same cell. **(C)** Typical APs of one atrial myocyte in absence (baseline) and presence of barium to block the native I_K1_. **(D)** Average AP parameters measured in four cells in presence of barium. AP parameters are normalized to the AP parameters measured under baseline conditions in the same cell. *p < 0.05, drug vs. baseline (paired *t*-test).

Fifth, the effects of the bee venom neurotoxin apamin (Banks et al., 1979), which is a highly potent and specific blocker of small conductance Ca^2+^-activated K^+^ channels (SK channels) (Xu et al., 2003; Yu et al., 2014), was tested at a concentration of 50 pM. Typical examples and the average effects of SK channel blockade on APs are shown in Figure 5A,B, respectively. Apamin significantly depolarized the RMP, significantly decreased APA and dV/dt_max_, and significantly increased APD_90_ with all three types of current injection. The effects of apamin did not differ significantly between the I_K1_ and constant current injection approaches.

#### Barium

Finally, we tested the effects of 1 mM Ba^2+^, which completely blocks inward rectifier channels (Schram et al., 2003), to find out whether I_K1_ is still functional in the initially depolarized cells. Figure 5C shows typical AP recordings before and after the addition of Ba^2+^ to the extracellular solution; Figure 5D summarizes the average AP changes. As illustrated, Ba^2+^ significantly depolarized the RMP, significantly decreased APA and dV/dt_max_, and induced significant prolongation of the APD_90_. The effects were similar with all three types of current injection.

## Discussion

### Overview

In accordance with previous studies, we here observed a wide range of RMP values in single human LAA myocytes following isolation. In the depolarized cells (RMP positive to −60 mV), RMP could be set at −80 mV using I_K1_ or constant current injection, and consequently APs could be evoked upon stimulation. The AP morphology of cells with current injection was quite similar to that of regular non-depolarized cells, especially when using I_K1_ with “Bett” characteristics, i.e., with moderate rectification. APs evoked using constant current or I_K1_ injection were still sensitive to all tested drugs (lidocaine, nifedipine, low dose of 4-AP, E-4031, apamin and barium), suggesting that the major ionic currents of the atrial cells were still functional in the initially depolarized myocytes. However, drug effects, especially those related to APD, were in some cases dependent on the current injection approach used.

### Current Injection Facilitates Action Potential Recordings From Human Atrial Myocytes

In ≈75% of the freshly isolated human atrial myocytes, we found an RMP positive to −60 mV (Figure 2A). While this is generally observed with patch clamp methodology in this cell type (Amos et al., 1996; Schreieck et al., 2000; Loose et al., 2014), the exact reason for the depolarized state of the RMP still remains to be resolved. It may be intrinsic to human LAA myocytes (Mary-Rabine et al., 1980; Escande et al., 1986), for example due to the presence of the hyperpolarization-activated inward current (I_f_) (Thuringer et al., 1992; Hoppe and Beuckelmann, 1998; Stillitano et al., 2013), the lower density of I_K1_ compared to ventricular myocytes (Varró et al., 1993), or the presence of an as yet unknown “native” depolarizing conductance. In addition, it may be introduced by the diseased state of the myocytes (Nattel et al., 2007), since the tissue in the present study is obtained from patients suffering from AF. However, depolarized cells are also frequently found in myocytes isolated from tissue obtained from patients undergoing aortocoronary bypass surgery and from donor hearts (Amos et al., 1996; Schreieck et al., 2000), suggesting that the diseased state is a less plausible explanation. In fact, I_K1_ is upregulated in response to AF (Van Wagoner et al., 1997; Bosch et al., 1999; Workman et al., 2001; Dobrev et al., 2005), suggesting a limited number of depolarized cells, which, however, was not the case, as shown in Figure 2A. It might also result from technical issues related to the patch clamp technique with imperfect seals resulting in “leak currents” that depolarize the RMP (Verkerk and Wilders, 2021). RMP depolarizations would be more pronounced in smaller cells, but in the present study non-depolarized and depolarized myocytes had a similar C_m_. Finally, effects from cell isolation procedures cannot be excluded (Varró et al., 2021). We here isolated myocytes enzymatically by the chunk method, and this method results in smaller I_K1_ densities compared to myocytes isolated using Langendorff perfusion (Hoshino et al., 2012).

The issue of the depolarized state of freshly isolated native human atrial myocytes is similar to that of hiPSC-CMs (Veerman et al., 2015), a relatively new and very promising model to test drugs (Magdy et al., 2018) and genetic disorders (Hoekstra et al., 2012; Brandão et al., 2017) in a human background. Recently, we and others have developed a dynamic clamp method to overcome the depolarized state of hiPSC-CMs [for reviews, see Ortega et al. (2018) and Verkerk and Wilders (2021)]. In the present study, we have extended the applicability of dynamic clamp to native human LAA myocytes of AF patients (Figure 2). We found that a stable RMP of −80 mV in our myocytes was achieved with ≈0.7 pA/pF repolarizing current. This amount of current is largely similar to the 0.3–1.2 pA/pF mentioned in, or calculated from, other studies (Bénardeau et al., 1996; Workman et al., 2001; Limberg et al., 2011; Workman et al., 2012) in human right atrial appendage myocytes. Using I_K1_ or constant current injection, APs could be evoked upon stimulation. The AP upstroke velocity, dV/dt_max_, was high, demonstrating that the AP upstroke is due to activation of I_Na_ (Berecki et al., 2010). The AP morphology was largely comparable to the so-called “Type A” and “Type 3” APs, as defined by Bénardeau et al. (1996) and Dawodu et al. (1996), respectively. Thus, the APs had a fast phase 1 repolarization, without a dome, and a plateau at around −20 mV. Other AP types were not observed, consistent with studies of Workman and colleagues (Workman et al., 2001; Workman et al., 2012). The exact reason for the notable finding of Type A/Type 3 APs is unknown. We exclude the dynamic clamp, or the use of the originally depolarized cells, as mechanism because it was also found when injecting Kir2.1 current, which lacks a repolarizing current at positive potentials, as well as in non-depolarized cells, without injection of any repolarizing current. It also may be related to experimental techniques. Here, we have used the perforated patch clamp technique for the AP measurements, while many studies employed the ruptured patch technique, where the pipette solution frequently contains EGTA to buffer intracellular Ca^2+^. Consequently, Ca^2+^ modulation of various ion channels and exchangers may be overlooked using the latter approach (Hume and Leblanc, 1988; Koivumäki et al., 2011). This may be of particular relevance for SK channels, the expression of which is increased during the early stages of AF (Ozgen et al., 2007; Qi et al., 2014). Moreover, effects of regional differences and heterogeneity in AP morphology in human atria (Gelband et al., 1972; Gong et al., 2008) may also contribute substantially to our primary finding of Type A/Type 3 APs. Finally, chronic treatment of AF patients with Ca^2+^ antagonists is known to depress I_Ca,L_, resulting in shorter APs with suppressed plateau amplitudes (Le Grand et al., 1991), but in our study just one out of 8 patients was treated with Ca^2+^ antagonists (Table 1).

In the present study, we used two different I_K1_ equations as well as a constant current to set the RMP at a non-depolarized level, which made AP elicitation possible. We found that the APD and AP plateau potential were dependent on the approach of current injection. In the case of the Kir2.1 I_K1_, which exhibits strong rectification (Figure 1, inset), the outward current at the AP plateau level is negligible (Figure 2C, blue lines). With the Bett I_K1_, which has a moderate rectification consistent with a more atrial-like I_K1_ (Bett et al., 2013), the outward current at the plateau level is no longer negligible (Figure 2C, green lines) and consequently shortens the AP and lowers the AP plateau potential substantially (Figure 2D). The constant current injection results in an even larger outward current at the plateau level (Figure 2C, red lines), causing a substantial lowering of the plateau level and most pronounced APD shortening (Figure 2D). These findings indicate the importance of the amount of rectification and agree with previous findings in hiPSC-CMs and computer simulations using mathematical models of hiPSC-CMs and human atrial cells (Dhamoon et al., 2004; Meijer van Putten et al., 2015; Verkerk et al., 2017). With the Bett I_K1_, AP plateau potential and APD match closely with those of non-depolarized cells (Figure 2D), suggesting that this approach of dynamic clamp current injection is most suitable to overcome the depolarized state of freshly isolated human atrial myocytes.

### Drug-Induced Action Potential Changes and Comparison With Previous Studies

We have additionally tested various drugs to find out whether their modulatory effects could still be detected in the APs measured with constant and dynamic clamp injected currents. We have focused on well-established drugs with well-known effects on human atrial tissue and/or cells.

#### Lidocaine

We found a lidocaine-induced reduction in dV/dt_max_ in human atrial myocytes, consistent with findings using sharp microelectrode measurements in human atrial fibers (Lauribe et al., 1989) and in agreement with the blockade of peak I_Na_ by lidocaine in human atria (Jia et al., 1993). We also observed a decrease of APD in response to lidocaine, which confirms previous findings in human atrial fibers (Lauribe et al., 1989). The AP shortening is likely due to blockade of the slowly inactivating component of I_Na_, in combination with a decrease in I_Na_ window current caused by a hyperpolarizing shift in I_Na_ voltage dependency (Jia et al., 1993). A lidocaine-induced AP shortening is also found in dog (Burashnikov et al., 2007), but is in contrast with findings in guinea pig, rabbit, and rat, where no effect (Betancourt and Dresel, 1979; Shirayama et al., 1991; Goineau et al., 2012) or even an increase in APD (Goldberg and Roberts, 1981) was observed. These contradictory findings in various species clearly highlight the need to validate drugs in a human myocyte setting.

While the effect on dV/dt_max_ was relatively mild, the severe APD decrease observed upon lidocaine treatment might be explained by the voltage dependency of lidocaine-induced I_Na_ blockade, with a preference of blockade at more positive potentials (Bean et al., 1983; Jia et al., 1993). In our experiments, we did not find a significant difference in lidocaine effects using the various current injections. Previously it was found that the effect of lidocaine on dV/dt_max_ is more pronounced in the setting of longer APs (Burashnikov et al., 2015), but apparently the APD differences due to the various current injections (Figure 2D) were too small to obtain a detectable effect in upstroke velocities of APs stimulated at 1 Hz.

#### Nifedipine

In our patch clamp studies, nifedipine shortened the APD, which is in agreement with previous findings in human atrial myocytes (Li and Nattel, 1997; Van Wagoner et al., 1999; Workman et al., 2001). The shortening was smaller compared to these previous studies, but that is likely related to the used concentrations. We used 0.1 µM nifedipine, which is close to the IC_50_ (Eroglu et al., 2020), while a concentration with almost complete I_Ca,L_ blockade was used in the previous studies. One should note that a nifedipine-induced decrease in I_Ca,L_ may also influence intracellular Ca^2+^ dynamics and consequently the sodium-calcium exchange current (I_NCX_), which has a relatively high impact on atrial APs due to its high expression level and the rather negative plateau of the atrial AP (Bénardeau et al., 1996; Carmeliet, 2004). Thus the AP shortening in response to nifedipine is likely not only attributable to reduced I_Ca,L_, but also due to a reduction in I_NCX_ (Bénardeau et al., 1996).

The effect of nifedipine on APD was more pronounced using the dynamic clamp-injected Kir2.1 I_K1_ compared to the Bett and constant current injections. We speculate that this relates to the distinct AP morphologies induced by the different current injection strategies, with a significantly longer APD obtained with Kir2.1 I_K1_ injection (Figure 2D). Previous studies revealed that drugs modulating APD have an augmented effect in cells with longer APs (Wu et al., 2008; Bárándi et al., 2010; Matsa et al., 2011). This can be explained by the lower net repolarizing current in longer APs, making such APs more sensitive to any change in repolarizing and depolarizing current (Gaur et al., 2020). Indeed, our experiments demonstrate that depending on the current injection approach used, drug effects can be more or less pronounced.

#### 4-Aminopyridine

A low dose of the selective I_Kur_ blocker 4-AP (Wang et al., 1993) significantly prolonged APD_20_, consistent with previous findings in isolated human atrial myocytes of patients undergoing coronary artery bypass surgery (Wang et al., 1993; Li et al., 2008) and in human right atrial trabeculae from patients in sinus rhythm and chronic AF (Wettwer et al., 2004). We also found a significant APD_50_ prolongation, consistent with the study of Li and colleagues (Li et al., 2008), and this prolongation was even more pronounced with the Kir2.1 I_K1_ injection. In our experiments, I_Kur_ blockade by 4-AP also resulted in a significant APD_90_ prolongation when the Kir2.1 I_K1_ injection was used, but not when the Bett or constant current injection was applied. Variable 4-AP effects on APD_90_ are also reported by others. Li et al. (2008) found no significant change in APD_90_, but Wang et al. (1993) found a 66% prolongation and Wettwer et al. (2004) reported a 7% increase in tissue of AF patients. In tissue of patients in sinus rhythm, however, the APD_90_ was shortened in response to 4-AP (Wettwer et al., 2004). Underlying disease with a consequent more positive AP plateau potential in AF tissue is thought to be responsible for these variable findings (Wettwer et al., 2004). Differences in AP plateau potential may also be an explanation for our distinct APD_90_ findings, with a prolongation only found with Kir2.1 I_K1_ injection, which typically has the largest AP plateau potential amplitude, PlatA (Figure 2D).

#### E-4031

In our experiments, we found that E-4031 prolonged the APD_50_ and APD_90_. This contrasts with previous findings in human atrial myocytes from patients in sinus rhythm (Wettwer et al., 2004; Skibsbye et al., 2014), although Wettwer and colleagues did mention a tendency to AP prolongation. The reason underlying this apparent discrepancy is unclear, but may be consequent to the diseased state of our cells and/or the use of initially depolarized AF cells in the present study. In addition, it might be related to differences in AP morphology because I_Kr_ channel activation during relatively short atrial APs with negative plateau phase will be far from complete (Carmeliet, 2004). Indeed, we found that the AP prolonging effect of E-4031 at 50% repolarization was more pronounced using the Kir2.1 I_K1_, likely because there is more time for channel activation in the setting of the relatively long APD_50_ associated with the Kir2.1 I_K1_ (Figure 2D). This, in combination with the lower net current at the plateau of the Kir2.1 I_K1_ APs, likely makes the effects of drugs that modulate the plateau current more easily detectable.

#### Apamin

Apamin did not affect APD_50_, but prolonged APD_90_ consistent with previously observed SK blockade effects in human single atrial myocytes and tissue (Xu et al., 2003; Skibsbye et al., 2014). In addition, we found that apamin depolarized the RMP, and decreased APA and dV/dt_max_ in agreement with NS8593-induced SK current reduction measured with microelectrodes in human atrial tissue (Skibsbye et al., 2014). Since the used apamin concentration does not block I_Na_ (Yu et al., 2014), the decreased dV/dt_max_ observed by us is likely the consequence of the RMP depolarization resulting in more inactivated I_Na_ channels (Berecki et al., 2010). The latter could also explain the observed decrease in APA. Apamin-induced AP prolongation has also been observed in mice (Xu et al., 2003), but not in dog and rat (Nagy et al., 2009), further emphasizing the need for experiments on human cells and tissue. In the present study, we have used cells from paroxysmal AF patients and there is growing evidence that SK channels may contribute to AF-induced APD abbreviation. Some laboratories report upregulation of SK channel expression in chronic AF, but this is not a consistent finding [Peyronnet and Ravens (2019) and Shamsaldeen et al. (2019), and primary references cited therein]. Therefore, we cannot exclude that the apamin-induced effects we observed are influenced by the diseased state of the tissue used.

#### Barium

We here demonstrate a small, but significant RMP depolarization upon application of Ba^2+^, which is consistent with previous findings in human right atrial appendage (Escande et al., 1986). This indicates that the native I_K1_ was still functionally present in the initially depolarized cells and that the injected current does not obscure changes in I_K1_ completely. However, spontaneous activity was not observed in the measured cells, as occurred in a computer model of a guinea pig ventricular myocyte upon an 81% decrease in I_K1_ (Silva and Rudy, 2003). On the other hand, the application of 0.05–5 mM Ba^2+^ usually did not induce automatic activity in single ventricular myocytes obtained from adult guinea pig hearts (Imoto et al., 1987). Anyhow, one should keep in mind that drug effects on the native I_K1_ will be masked by the *in silico* injection of I_K1_ to a certain extent, simply because the *in silico* I_K1_ is not affected by the drug. The observed RMP depolarization may also lead to inactivation of I_Na_ channels and thus underlie the observed decrease in APA and dV/dt_max_ (Berecki et al., 2010). Additionally, we here observed a substantial Ba^2+^-induced increase in APD_90_ (Figure 5C,D), consistent with previous findings in human right atrial appendage and papillary muscle (Escande et al., 1986; Jost et al., 2013). The effects of I_K1_ blockade on the APs were independent of the used current injections.

### Limitations

We demonstrated that the APs of initially depolarized atrial myocytes, with three different types of current injection to arrive at a stable RMP near −80 mV, were sensitive to all drugs tested. This indicates that the major ionic currents were still functional in the initially depolarized cells. Drugs were not tested on intrinsically non-depolarized cells because of the limited number of such cells. Therefore, we cannot exclude that drug effects differ between initially depolarized myocytes on the one hand and intrinsically non-depolarized cells on the other hand. Frequency dependence was not tested in initially depolarized myocytes due to the already lengthy experimental protocol of the three current injection approaches in combination with the drug effect evaluations. Further studies are required to test whether the frequency dependencies of the initially depolarized myocytes and the non-depolarized ones are similar.

In our patch clamp experiments, we injected an *in silico* I_K1_ or constant current of ≈0.7 pA/pF to obtain a stable RMP near −80 mV in LAA myocytes of AF patients. It has been consistently demonstrated, in both animal models and humans, that I_K1_ is upregulated during AF (Van Wagoner et al., 1997; Bosch et al., 1999; Workman et al., 2001; Dobrev et al., 2005), resulting in a 4–6 mV RMP hyperpolarization in AF tissue. Thus, if myocytes of patients in sinus rhythm would be included, the I_K1_ density must likely be adapted with also consequent effects on the AP repolarization. Nevertheless, this limitation could also be considered as strength of the dynamic clamp to systematically study the effects of remodeled I_K1_ in human AF, but further studies are needed to address this issue in detail.

## Conclusion

Patch-clamp recordings of APs from human atrial myocytes can be optimized through dynamic clamp in order to provide these cells with a substantial I_K1_ resulting in a close-to-physiological RMP. The APs measured using moderate I_K1_ rectification (“Bett” I_K1_) matched closely those of non-depolarized cells, but effects of AP prolonging or shortening drugs are more pronounced using the dynamic clamp-injected I_K1_ with strong I_K1_ rectification (“Kir2.1” I_K1_) for drugs blocking I_Ca,L_, I_Kur_, or I_Kr_. Dynamic clamp therefore constitutes an appropriate tool for studying AP properties of human atrial myocytes, especially for the purpose of testing novel antiarrhythmic drugs.

## Data Availability

The raw data supporting the conclusions of this article will be made available by the authors, without undue reservation.

## Glossary

4-AP: 4-aminopyridine
ACE: angiotensin-converting enzyme
AF: atrial fibrillation
AP: action potential
APA: action potential amplitude
APD: action potential duration
APD_20_: action potential duration at 20% repolarization
APD_50_: action potential duration at 50% repolarization
APD_90_: action potential duration at 90% repolarization
C_m_: cell membrane capacitance
dV/dt_max_: maximum action potential upstroke velocity
E_K_: potassium equilibrium potential
hiPSC-CMs: human induced pluripotent stem cell-derived cardiomyocytes
IC_50_: half-maximal inhibitory concentration
I_Ca,L_: L-type Ca^2+^ current
I_f_: hyperpolarization-activated inward current
I_in_: injected current
I_K1_: inward rectifier K^+^ current
I_Kr_: rapid component of the delayed rectifier K^+^ current
I_Kur_: ultra-rapid component of the delayed rectifier K^+^ current
I_Na_: fast sodium current
I_NCX_: sodium-calcium exchange current
I_stim_: stimulus current
I_to1_: transient outward K^+^ current
LAA: left atrial appendage
MEA: multi-electrode array
PlatA: amplitude of the action potential plateau
RMP: resting membrane potential
RT-Linux: Real-Time Linux
SK channels: small conductance Ca^2+^-activated K^+^ channels
V_m_: membrane potential
